# Self-Learning Variable Structure Control for a Class of Sensor-Actuator Systems

**DOI:** 10.3390/s120506117

**Published:** 2012-05-10

**Authors:** Sanfeng Chen, Shuai Li, Bo Liu, Yuesheng Lou, Yongsheng Liang

**Affiliations:** 1 Key Lab of Visual Media Processing and Transmission, Shenzhen Institute of Information Technology, Shenzhen 518029, Guangdong, China; E-Mails: chensanf@sziit.com.cn (S.C.); liangys@sziit.com.cn (Y.L.); 2 Department of Electrical and Computer Engineering, Stevens Institute of Technology, Hoboken, NJ 07030, USA; E-Mail: sam.shuai.li@gmail.com; 3 Department of Computer Science, University of Massachusetts, Amherst, MA 01003, USA; 4 School of Mechatronics and Information, Yiwu Industrial and Commercial College, Yiwu 322000, Zhejiang, China; E-Mail: lusion@mail.ustc.edu.cn

**Keywords:** sensor-actuator system, principle of optimality, Bellman equation, variable structure control, self-learning

## Abstract

Variable structure strategy is widely used for the control of sensor-actuator systems modeled by Euler-Lagrange equations. However, accurate knowledge on the model structure and model parameters are often required for the control design. In this paper, we consider model-free variable structure control of a class of sensor-actuator systems, where only the online input and output of the system are available while the mathematic model of the system is unknown. The problem is formulated from an optimal control perspective and the implicit form of the control law are analytically obtained by using the principle of optimality. The control law and the optimal cost function are explicitly solved iteratively. Simulations demonstrate the effectiveness and the efficiency of the proposed method.

## 1. Introduction

With the development of mechatronics, automatic systems consisting of sensors for perception and actuators for action are more and more widely used in applications [1–4]. Besides the proper choices of sensors and actuators and an elaborate fabrication of mechanical structures, the control law design also plays a crucial role in the implementation of automatic systems especially for those with complicated dynamics. For most mechanical sensor-actuator systems, it is possible to model them in Euler-lagrange equations [4,5]. In this paper, we are concerned with the sensor-actuator systems modeled by Euler-lagrange equations.

Due to the importance of Euler-lagrange equations in modeling many real sensor-actuator systems, much attention has been paid to the control of such kind systems. According to the type of constraints, the Euler-lagrange system can be categorized into Euler-lagrange system without nonholonomic constraints (e.g., fully-actuated manipulator [6,7], omni-directional mobile robot [8]), and the system subject to nonholonomic constraint [9] (e.g., the cart-pole system [10], the under-actuated multiple body system [11]). For Euler-lagrange system without nonholonomic constraints, the dimension of inputs are often equal to the dimension of output and the system are often able to be transformed into a double integrator system by employing feedback linearization [12]. Other methods, such as control Lyapunov function method [13], passivity based method [14], optimal control method [15], etc., are also successfully applied to the control of Euler-lagrange system without nonholonomic constraints. In contrast, as the dimension of inputs is lower than that of outputs, it is often impossible to directly transform the Euler-lagrange system subject to nonholonomic constraints to a linear system and thus feedback linearization fails to stabilize the system. To tackle the difficulty, variable structure control based method [16], backstepping based control [17], optimal control based method [18], discontinuous control method [19], etc., are widely investigated and some useful design procedures are proposed. However, due to the inherent nonlinearity and nonholonomic constraints, most existing methods [16–19] are strongly model dependent and the performance are very sensitive to model errors. Inspired by the success of human operators for the control of Euler-lagrange systems, various intelligent control strategies, such as fuzzy logic [20], neural networks [21], evolutionary algorithms [22], to name a few of them, are proposed to solve the control problem of of Euler-lagrange systems subject to nonholonomic constraints. As demonstrated by extensive simulations, these type of strategies are indeed effective to the control of Euler-lagrange systems subject to nonholonomic constraints. However, rigorous proof on the stability are difficult for this type of methods and there may exist some initializations of the state, from which the system cannot be stabilized.

In this paper, we propose a self-learning control method applicable to Euler-lagrange systems. In contrast to existing work on intelligent control of Euler-lagrange systems, the stability of the close loop system with the proposed method is proven in theory. On the other hand, different from model based design strategies, such as backstepping based design [17], variable structure based design [16], *etc*., the proposed method does not require information of the model parameters and therefore is a model independent method. We formulate the problem from an optimal control perspective. In this framework, the goal is to find the input sequence to minimize the cost function defined on infinite horizon under the constraint of the system dynamics. The solution can be found by solving a Bellman equation according to the principle of optimality [23]. Then an adaptive dynamic programming strategy [24–26] is utilized to numerically solve the input sequence in real time.

The remainder of this paper is organized as follows: in Section 2, preliminaries on Euler-lagrange systems and variable structure control are given briefly. In Section 3, the problem is formulated as a constrained optimization problem and the critic model and the action model are employed to approximate the optimal mappings. The control law is then derived in Section 4. In Section 5, simulations are given to show the effectiveness of the proposed method. The paper is concluded in Section 6.

## 2. Preliminaries on Variable Structure Control of the Sensor-Actuator System

In this paper, we are concerned with the following sensor-actuator system in the Euler-Lagrange form,

(1) $$D(q)\ddot{q}+C(q,\dot{q})\dot{q}+\phi (q)=u$$

where *q* ∈ ℝ*^n^, D*(*q*) ∈ ℝ^n×^*^n^* is the inertial matrix, *C*(*q,q˙*) ∈ ℝ*^n^*^×^*^n^, ϕ*(*q*) ∈ ℝ*^n^* and *u* ∈ ℝ*^n^*. Note that the inertial matrix *D*(*q*) is symmetric and positive definite. There are three terms on the left side of the above equation. The first term involve the inertial force in the generalized coordinates, the second one models the Coriolis force and friction, the values of which depend on *q̇* and the third one is the conservative force, which is in correspondence to the potential energy. The control force *u* applied on the system drives the variation of the coordinate *q*. It is also noteworthy that we assume the dimension of *u* is equal to that of *q* here. This definition also admits the case for *u* with lower dimension than that of *q* by imposing constraints to *u*, e.g., the constraint *u =* [*u*_1_,*u*_2_, *…,u_n_*] with *u*_1_ = 0 restricts *u* in *a n* – 1 dimensional space. Defining state variables *x*_1_ = *q* and *x*_2_ *= q*, the Euler-Lagrange Equation (1) can be put into the following state-space form:

(2) $$\begin{array}{lll} {\dot{x}}_{1} & = & x_{2}\\ {\dot{x}}_{2} & = & -D^{-1}(x_{1})\left(u+C(x_{1},x_{2})x_{2}+\phi (x_{1})\right) \end{array}$$

Note that the matrix *D*(*x*_1_) is invertible as it is positive definite. The control objective is to asymptotically stabilize the Euler-Lagrange system (2), *i.e.*, design a mapping (*x*_1_,*x*_2_) *→ u* such that *x*_1_ → 0 and *x*_2_ → 0 when time elapses.

As an effective design strategy, variable structure control finds applications in many different type of control systems including the Euler-Lagrange system. The method stabilizes the dynamics of a nonlinear system by steering the state to a elaborately designed sliding surface, on which the state inherently evolves towards the zero state. Particularly for the system (2), we define *s = s*(*x*_1_,*x*_2_) as follows:

(3) $$s=c_{0}x_{1}+x_{2}$$

where *c*_0_ *>* 0 is a constant. Note that *s = c*_0_*x*_1_ + *x*_2_ *=* 0 together with the dynamics of *x*_1_ in Equation (2) gives the dynamics of *x*_1_ as *ẋ_1_*) *=* –*c*_0_*x*_1_ for *c*_0_ *>* 0. Clearly, *x*_1_ asymptotically converges to zero. Also we know *x*_2_ = 0 when *x*_1_ = 0 according to *s =* c_0_*x*_1_ + *x*_2_ = 0. Therefore, we conclude the states x_1_, *x*_2_ on the sliding surface *s =* 0 for *s* defined in Equation (3) converge to zero with time. With this property of the sliding surface, a control law driving the states to *s =* 0 definitely grantees the ultimate convergence to the zero states. Accordingly, the stabilization of the system can be realized by controlling *s* to zero. To reach this goal, a positive definite control Lyapunov function *V*(*s*), e.g., *V*(*s*) *= s*^2^, is often used to design the control law. For stability consideration, the time derivative of *V*(*s*) is required to be negative definite. In order to guarantee the negative definiteness of the time derivative of *V*(*s*), exact information about the system dynamics (2) is often necessary, which results in the model based design strategies.

About the Euler-Lagrange Equation (1) for modeling sensor-actuator systems, we have the following remark:

**Remark 1** *In this paper, we are concerned with the class of sensor-actuator systems modeled by the Euler-Lagrange Equation (1). Actually, the dynamics of mechanical systems can be described by the Euler-Lagrange equation according to the rigid body mechanics [4,5], which is essentially equivalent to Newton's laws of motion. Therefore, mechanical sensor-actuator system can be modeled by Equation (1). In this regard, the Euler-Lagrange equation employed in the paper models a general class of sensor-actuator systems.*

## 3. Problem Formulation

Without losing generality, we stabilize the system (1) by steering it to the sliding surface *s* = 0 with *s* defined in Equation (3). Different from existing model based design procedures, we design a self-learning controller, which does not require accurate knowledge about *D*(*q*), *C*(*q,q̇*) and *ϕ*(*q*) in Equation (1). In this section, we formulate such a control problem from the optimal control perspective.

In this paper, we set the origin as the desired operating point, *i.e.*, we consider the problem of controlling the state of the system (1) to the origin. For the case with other desired operating points, the problem can be equivalently transformed to the one with the origin as the operating point by shifting the coordinates. At each sampling period, the norm of *s = c*_0_*x*_1_ + *x*_2_, which measures the distance from the desired sliding surface *s* = 0, can be used to evaluate the one step performance. Therefore, we define the following utility function associated with the one-step cost at the *i*th sampling period,

(4) $$U_{i}=U(s)$$

with

(5) $$U(s)=\{\begin{array}{cc} 0 & \left|s_{1}\right|<\delta_{1},\left|s_{2}\right|<\delta_{2},\ldots ,\left|s_{n}\right|<\delta_{n}\\ 1 & \text{otherwise} \end{array}$$

where *s* is defined in Equation (3) and *s =* [*s*_1_,*s*_2_, *…, s_n_*]*^T^*, |*s_i_*| denotes the absolute value of the *i*th component of the vector *s*, the parameter *δ_i_>* 0 for *i* = 1, 2,…, *n*. At each step, there is a value *U_i_* and the total cost starting from the *k*th step along the infinite time horizon can be expressed as follows,

(6) $$J_{k}=J(x(k),\bar{u}(k))=\sum_{i=k}^{\infty }\gamma^{i-k}U_{i}$$

where *x*(*k*) is the state vector of system (1) sampled at the *k*th step with $x(k)={\left[x_{1}^{T}(k),x_{2}^{T}(k)\right]}^{T}$ γ is the discount factor with 0 < γ < 1, *ū*(*k*) *=* (*u_k_,u_k_+*1,…,*u*_∞_) is the control sequence starting from the *k*th step. Note that for the deterministic system (1), the preceding states after the *k*th step are determined by *x*(*k*) and the control sequence *ū_k_*. Accordingly, *J_k_* is a function of *x*(*k*) and *ū*(*k*) with *J_k_* = *J*(*x*(*k*), *ū*(*k*)). Also note that both the cost function *J_k_* and the utility function *U_k_* are defined based on the discrete samplings of the continuous system (1). Now, we can define the problem of controlling the sensor-actuator system (1) in this framework as follows,

(7a) $$\underset{u(0),u(1),\ldots ,u(\infty )\in \Omega }{\min}J_{0}=\sum_{i=0}^{\infty }\gamma^{i}U_{i}$$

subject to:

(7b) $$\{\begin{array}{lll} {\dot{x}}_{1}(t) & = & x_{2}(t)\\ {\dot{x}}_{2}(t) & = & -D^{-1}(t)(x_{1}(t))\;\left(u(t)+C(x_{1}(t),x_{2}(t))x_{2}(t)+\phi (x_{1}(t))\right) \end{array}$$

(7c) u(t)=u(i)foriτ≤t<(i+1)τ

where *U_i_* is defined by Equations (4) and (5), τ > 0 is the sampling period, the set Ω defines the feasible control actions, *J*_0_ is the cost function for *k* = 0 in Equation (6). It is worth noting that *J*_0_ is a function of *ū*(0) *=* (*u*_0_, *u*_1_,…, *u*_∞_) and *x*(0) according to Equation (6). The optimization in Equation (7) is relative to *ū*(0) with a given initial state *x*(0). Also note that in the optimization problem in Equation (7), the decision variable *u*(0),*u*(1), …,*u*(∞) are defined in every sampling period. The control action keeps the value in the duration of two consecutive sampling steps. This formulation is consistent with the real implementations of digital controllers.

**Remark 2** *There are infinitely many decision variables, which are u(0), u(1), …, u(∞), in the optimization problem in Equation (7). Therefore, this is an infinite dimensional problem. It cannot be solved directly using numerical methods. Conventionally, such kind of problem is often solved by using a finite dimensional approximation [27]. In addition, note that the dynamic model of the system appears in the optimization problem in Equation (7) and it will also show up in the finite dimensional relaxation of the problem, which means the resulting solution requires model information and thus is also model-dependent. In contrast, in this paper we investigate the model-independent variable structure control of sensor-actuator systems on the infinite time horizon.*

## 4. Model-Free Control of the Euler-Lagrange System

In this section, we present the strategy to solve the constrained optimization problem efficiently without knowing the model information of the chaotic system. We first investigate the optimality condition of Equation (7) and present an iterative procedure to approach the analytical solution. Then, we analyze the convergence of the iterative procedure and the stability with the derived control strategy.

### 4.1. Optimality Condition

Denoting J* the optimal value to the optimization problem in Equation (7), *i.e.*,

(8) $$\begin{array}{cc} J^{*}=\min_{u(0),u(1),\ldots ,u(\infty )\in \Omega } & J_{0} \end{array}$$

subject to: (7b); (7c)

According to the principle of optimality [23], the solution of Equation (7) satisfy the following Bellman equation:

(9) $$J*(y)=\underset{u_{k}\in \Omega }{\min}(U_{k}+\gamma J*(z))\forall x,\forall k=0,1,2,\ldots$$

where *z* is the solution of Equation (7b) at *t = k* + 1 with *x*(*k*) *= y* and the control action *u*(*t*) *= u_k_* for *kτ ≤ t <* (*k* + 1)τ. Without introducing confusion, we simply write Equation (9) as follows

(10) $$J*=\min(U_{k}+\gamma J*)$$

Define the Bellman operator *ℬ* relative to function *h*(*z*) as follows

(11) $$ℬh(z)=\min(U_{k}+\gamma h(z))$$

Then, the optimality condition in Equation (10) can be simplified into the following with the Bellman operator,

(12) J∗=ℬJ∗

Note that the function *U_k_* is implicitly included in the Bellman operator. The Equation (12) constitutes the optimality condition for problem in Equation (7). It is difficult to solve the explicit form of J* analytically from Equation (9). However, it is possible to get the solution by iterations. We use the following iterations to solve J*,

(13) $$\hat{J}(n+1)=ℬ\hat{J}(n)$$

subject to: (7b); (7c)

The control action keeps constant in the duration between the *k*th and the *k* + 1th step, i.e., u*(*t*) *=* $u_{k}^{*}$ for *kτ ≤ t <* (*k* + 1)τ. $u_{k}^{*}$ can be obtained from Equation (9) based on Equation (13),

(14) $$u_{k}^{*}={\text{argmin}}_{u_{k}\in \Omega }(U_{k}+\gamma J*)$$

### 4.2. Approximating the Action Mapping and the Critic Mapping

In the previous sections, the iteration (13) is derived to calculate J* and the optimization (14) is obtained to calculate the control law. The iteration to approach J* and the optimization to derive u* have to be run in every time step in order to obtain the most up-to-date values. Inspired by the learning strategies widely studied in artificial intelligence [26,28], a learning based strategy is used in this section to facilitate the processing. After a enough long time, the system is able to memorize the mapping of J* and the mapping of u*. After this learning period, there will be no need to repeat any iterations or optimal searching, which will make the strategy more practical.

Note that the optimal cost J* is a function of the initial state. Counting the cost from the current time step, J* can also be regarded as a function of both the current state and the optimal action at current time step according to Equation (10). Therefore, *ĵ*(*n*), the approximation of J*, can also be regarded as a function relative to the current state and the current optimal input. As to the optimal control action u*, it is a function of the current state. Our goal in this section is to obtain the mapping from the current state and the current input to *ĵ* (*n*) and the mapping from the current state to the optimal control action u* using parameterized models, denoted as the critic model and the action model, respectively. Therefore, we can write the critic model and the action model as *J_n_*( $u_{n}^{*}$ *x_n_, W_c_*) and $u_{n}^{*}$ (*x_n_, W_a_*) respectively, where *W_c_* is the parameters of the critic model and *W_a_* is the parameters of the action model.

In order to train the critic model with the desired input-output correspondence, we define the following error at time step *n* + 1 to evaluate the learning performance,

(15) $$\begin{array}{cc} e_{c}(n+1) & =ℬ\hat{J}(n)-\hat{J}(n+1)\\ E_{c}(n+1) & =\frac{1}{2}e_{c}^{2}(n+1) \end{array}$$

Note that *Bĵ*(*n*) is the desired value of *ĵ*(*n +* 1) according to Equation (13). Using the back-propagation rule, we get the following rule for updating the weight *W_c_* of the critic model,

(16) $$\begin{array}{ll} & W_{c}(n+1)\\ = & W_{c}(n)+\delta W_{c}(n)\\ = & W_{c}(n)-l_{c}(n)\frac{\partial E_{c}(n)}{\partial W_{c}(n)}\\ = & W_{c}(n)-l_{c}(n)\frac{\partial E_{c}(n)}{\partial \hat{J}(n)}\frac{\partial \hat{J}(n)}{\partial W_{c}(n)} \end{array}$$

where *l_c_*(*n*) is the step size for the critic model at the time step *n*.

As to the action model, the optimal control u* in Equation (14) is the one that minimizes the cost function. Note that the possible minimum cost is zero, which corresponds to the scenario with the state staying inside the desired bounded area. In this regard, we define the action error as follows,

(17) $$\begin{array}{cc} e_{a}(n) & ={\hat{J}}_{n}\\ E_{a}(n) & =\frac{1}{2}e_{a}^{2}(n) \end{array}$$

Then, similar to the update rule of *W_c_* for the critic model, we get the following update rule of *W_a_* for the action model,

(18) $$W_{a}(n+1)=W_{a}(n)-l_{a}(n)\frac{\partial E_{a}(n)}{\partial \hat{J}(n)}\frac{\partial \hat{J}(n)}{\partial u(n)}\frac{\partial u(n)}{\partial W_{a}(n)}$$

where *l_a_*(*n*) is the step size for the action model at the time step *n*.

Equations (16) and (18) update the critic model and the action model progressively. After *W_c_* and *W_a_* have learnt the model information by learning for a long enough time, their values can be fixed at the one obtained at the final step and no further learning is required any longer, which is in contrast to Equation (14) requiring to solve an optimization problem even after a long enough time.

## 5. Simulation Experiment

In this section, we consider the simulation implementation of the proposed control strategy. The dynamics given in Equation (1) model a wide class of sensor-actuator systems. Particularly, to demonstrate the effectiveness of the proposed self-learning variable structure method, we apply it to the stabilizations of a typical benchmark system: the cart-pole system.

**Figure 1.**
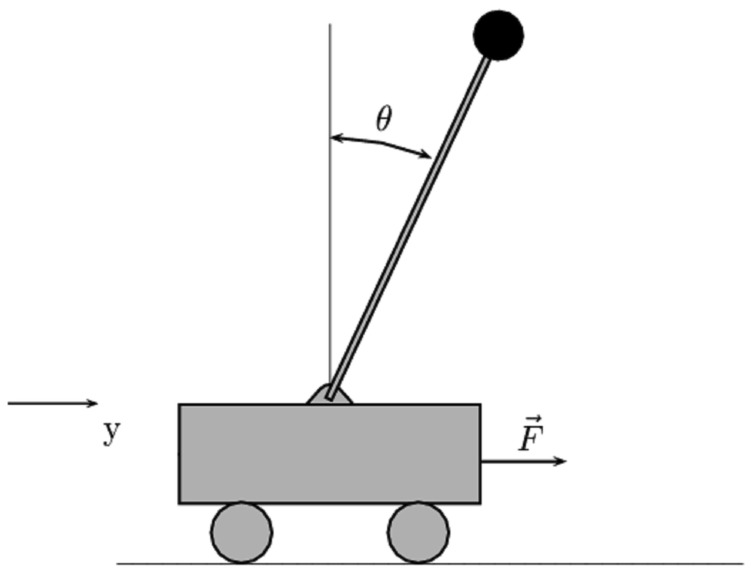
The cart-pole system.

The cart-pole system, as sketched in Figure 1, is a widely used testbed for the effectiveness of control strategies. The system is composed of a pendulum and a cart. The pendulum has its mass above its pivot point, which is mounted on a cart moving horizontally. In this part, we apply the proposed control method to the cart-pole system to test the effectiveness of our method.

### 5.1. The Model

The cart-pole model used in this work is the same as that in [29], which can be described as follows.

(19) $$\ddot{\theta }=\frac{g\sin\theta +\cos\theta [-F-ml{\dot{\theta }}^{2}\sin\theta +\mu_{c}\mathrm{sgn}(\dot{y})]-\frac{\mu_{p}\dot{\theta }}{ml}}{l\left(\frac{4}{3}-\frac{m\cos^{2}\theta }{m_{c}+m}\right)}$$

(20) $$\ddot{y}=\frac{F+ml[{\dot{\theta }}^{2}\sin\theta -\ddot{\theta }\cos\theta ]-\mu_{c}\mathrm{sgn}(\dot{y})}{m_{c}+m}$$

where

(21) $$\mathrm{sgn}(x)=\{\begin{array}{ccc} 1 & if & x>0\\ 0 & if & x=0\\ -1, & if & x<0 \end{array}$$

with the following values of the parameters:

*g*: 9.8 *m/s*^2^, acceleration due to gravity;
*m_c_*: 1.0 kg, mass of cart;
*m*: 0.1 kg, mass of pole;
*l*: 0.5 meter, half-pole length;
*μ_c_*: 0.0005, coefficient of friction of cart on track;
*μ_p_*: 0.000002, coefficient of friction of pole on cart;
*F*: ±10 Newtons, force applied to cart center of mass.

This system has four state variables: *y* is the position of the cart on track, *θ* is the angle of the pole with respect to the vertical position, and *ẏ* and *θ̇* are the cart velocity and angular velocity, respectively.

Define $A_{1}(\theta )=-\frac{l}{\cos\theta }\left(\frac{4}{3}-\frac{m\cos^{2}\theta }{m_{c}+m}\right)$, $A_{2}(\theta )=\frac{g\sin\theta }{\cos\theta }$, $A_{3}(\theta ,\dot{\theta })=ml\dot{\theta }\sin\theta +\frac{\mu_{p}}{\text{ml}\cos\theta }$, $A_{4}(\dot{y})=\frac{\mu_{c}\mathrm{sgn}(\dot{y})}{\dot{y}}$, *A*_5_ = *m_c_* + *m, A*_6_(*θ,θ̇*) *= ml*θ̇ *sinθ, A*_7_(*θ*) *=* –*ml cosθ*. With these notations, Equation (19) can be re-written as:

(22) $$\begin{array}{cc} A_{1}\ddot{\theta } & =F+A_{2}+A_{3}\dot{\theta }+A_{4}\dot{y}\\ \frac{A_{1}+A_{5}}{A_{1}+A_{7}}\ddot{y} & =F+\frac{A_{2}A_{7}}{A_{1}+A_{7}}+\frac{A_{1}A_{6}+A_{3}A_{7}}{A_{1}+A_{7}}\dot{\theta }+\frac{A1A_{4}+A_{4}A_{7}}{A_{1}+A_{7}}\dot{y} \end{array}$$

By choosing

$$\begin{array}{r} D=\left[\begin{array}{cc} A_{1} & 0\\ 0 & \frac{A_{1}A_{5}}{A_{1}+A_{7}} \end{array}\right],C=-\left[\begin{array}{cc} A_{3} & A_{4}\\ \frac{A_{1}A_{6}+A_{3}A_{7}}{A_{1}+A_{7}} & \frac{A_{1}A_{4}+A_{4}A_{7}}{A_{1}+A_{7}} \end{array}\right]\\ \phi =-\left[\begin{array}{c} A_{2}\\ \frac{A_{2}A_{7}}{A_{1}+A_{7}} \end{array}\right],q=\left[\begin{array}{c} \theta \\ y \end{array}\right],u=\left[\begin{array}{c} F\\ F \end{array}\right] \end{array}$$

the system of Equation (19) coincides with the model of Equation (1). Note that the input *u* in this situation is constrained in the set Ω = {*u =* [*u*_1_, u_2_]*^T^, u*_1_ *= u*_2_ ∈ ℝ}.

### 5.2. Experiment Setup and Results

**Figure 2.**
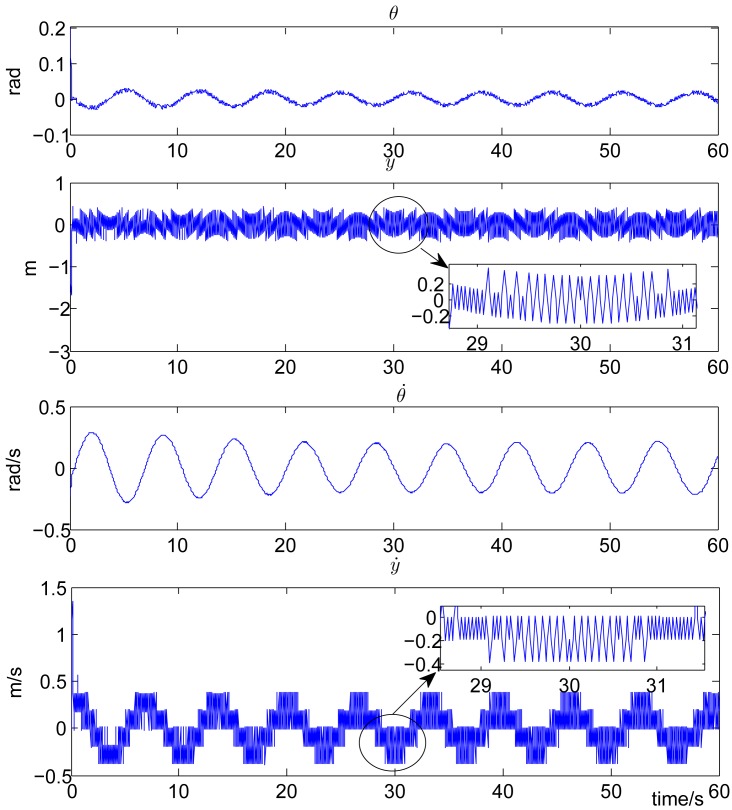
State profiles of the cart-pole system with the proposed control strategy.

In the simulation experiment, we set the discount factor γ = 0.95, the sliding surface parameter *k* = 10, *δ*_1_ = 2, *δ*_2_ = 24. The feasible control action set Ω in Equation (7) is defined as Ω = {*u =* [*u*_1_,*u*_2_]*^T^,u*_1_ ∈ ℝ,u_2_ ∈ ℝ,*u*_1_ = *u*_2_ = ±10 Newtons}. This definition corresponds to the widely used bang-bang control in industry. To make the output of the action model within the feasible set, the output of the action network is clamped to 10 if it is greater than or equal to zero and clamped to – 10 if less than zero. The sampling period τ is set to 0.02 seconds. Both the critic model and the action model are linearly parameterized. The step size of the critic model, which is *l_c_*(*n*) and that of the action model, which is *l_a_*(*n*) are both set to 0.03. Both the update of the critic model weight *W_c_* in Equation (16) and the update of the action model weight *W_a_* in Equation (18) last for 30 seconds. For the uncontrolled cart-pole system with *F* = 0 in Equation (19), the pendulum will fall down. The control objective is to stabilize the pendulum to the inverted direction (*θ =* 0). Time history of the state variables are plotted in Figure 2 for the system with the proposed self-learning variable structure control strategy. From this figure, it can be observed that *θ* is stabilized in a small vicinity around zero (with a small error of ±0.1 rads), which corresponds to the inverted direction.

## 6. Conclusions and Future Work

In this paper, the self-learning variable structure control is considered to solve a class of sensor-actuator systems. The control problem is formulated from the optimal control perspective and solved via iterative methods. In contrast to existing models, this method does not need pre-knowledge on the accurate mathematic model. The critic model and the the action model are introduced to make the method more practical. Simulations show that the control law obtained by the proposed method indeed achieves the control objective. Future work on this topic includes the theoretical proof of the convergence and exploration on the performance limit of the proposed strategy. Also, the control of other mechanical systems modeled by Euler-Lagrange system, such as manipulators *etc*., will be explored in our future work.

## References

[1] Isermann R. (1996). Modeling and design methodology for mechatronic systems. IEEE/ASME Trans. Mechatr..

[2] van de Panne M., Fiume E. Sensor-actuator networks.

[3] Liu B., Chen S., Li S., Liang Y. (2012). Intelligent control of a sensor-actuator system via kernelized least-squares policy iteration. Sensors.

[4] de Silva C. (2007). Sensors and Actuators: Control System Instrumentation.

[5] Beer F.P. (2003). Vector Mechanics for Engineers: Statics and Dynamics.

[6] Lewis F.L., Dawson D.M., Abdallah C.T. (2004). Manipulator Control Theory and Practice.

[7] Li S., Chen S., Liu B., Li Y., Liang Y. (2012). Decentralized kinematic control of a class of collaborative redundant manipulators via recurrent neural networks. Neurocomputing.

[8] Li S., Meng M.Q.H., Chen W. SP-NN: A novel neural network approach for path planning.

[9] Bloch A.M. (2003). Nonholonomic Mechanics and Control.

[10] Yu H., Liu Y., Yang T. Tracking control of a pendulum-driven cart-pole underactuated system.

[11] Seifried R. (2012). Two approaches for feedforward control and optimal design of underactuated multibody systems. Multibody Syst. Dynam..

[12] Isidori A. (1999). Nonlinear Control Systems II.

[13] Primbs J.A., Nevistic V., Doyle J.C. (2009). Nonlinear optimal control: A control lyapunov function and receding horizon perspective. Asian J. Control.

[14] Ortega R., Loria A., Nicklasson P.J., Sira-Ramirez H. (1998). Passivity-Based Control of Euler-Lagrange Systems.

[15] Azhmyakov V. (2007). Optimal control of mechanical systems. Diff. Equat. Nonlin. Mech..

[16] Huo W. (2008). Predictive variable structure control of nonholonomic chained systems. Int. J. Comput. Math..

[17] Dumitrascu B., Filipescu A., Minzu V., Filipescu A. Backstepping control of wheeled mobile robots.

[18] Hussein I.I., Bloch A.M. (2005). Optimal control of underactuated nonholonomic mechanical systems. IEEE Trans. Autom. Control..

[19] Pazderski D., Kozowski K., Krysiak B., Kozlowski K. (2009). Nonsmooth stabilizer for three link nonholonomic manipulator using polar-like coordinate representation. Robot Motion and Control.

[20] Cuesta F., Ollero A., Arrue B.C., Braunstingl R. (2003). Intelligent control of nonholonomic mobile robots with fuzzy perception. Fuzzy Sets Syst..

[21] Wai R.J., Liu C.M. (2009). Design of dynamic petri recurrent fuzzy neural network and its application to path-tracking control of nonholonomic mobile robot. IEEE Trans. Indust. Electr..

[22] Kinjo H., Uezato E., Duong S.C., Yamamoto T. (2009). Neurocontroller with a genetic algorithm for nonholonomic systems: Flying robot and four-wheel vehicle examples. Artif. Life Robot..

[23] Bertsekas D.P. (2005). Dynamic Programming and Optimal Control.

[24] Murray J.J., Cox C.J., Lendaris G.G., Saeks R. (2002). Adaptive dynamic programming. IEEE Trans. Syst. Man Cyber..

[25] Lewis F.L., Vrabie D. (2009). Reinforcement learning and adaptive dynamic programming for feedback control. IEEE Circuits Syst. Mag..

[26] Si J., Barto A., Powell W., Wunsch D. (2004). Handbook of Learning and Approximate Dynamic Programming.

[27] Mayne D.Q., Michalska H. (1990). Receding horizon control of nonlinear systems. IEEE Trans. Autom. Control.

[28] Bishop C.M. (2006). Pattern Recognition and Machine Learning.

[29] Si J., Wang Y.T. (2001). Online learning control by association and reinforcement. IEEE Trans. Neural Netw..

